# Paraneoplastic Scleroderma: A Case Report of Systemic Sclerosis in the Setting of Pulmonary Adenocarcinoma

**DOI:** 10.7759/cureus.36366

**Published:** 2023-03-19

**Authors:** Christopher Marsalisi, Avni Agrawal, Abhinav Karan, Zachary Chandler, Pramod Reddy

**Affiliations:** 1 Internal Medicine, University of Florida College of Medicine - Jacksonville, Jacksonville, USA; 2 Internal Medicine, University of Florida Health Jacksonville, Jacksonville, USA

**Keywords:** autoimmune rheumatic diseases, adenocarcinoma of the lung, paraneoplastic rheumatic syndrome, paraneoplastic syndromes, systemic scleroderma

## Abstract

A 42-year-old female with a past medical history significant for scleroderma and extensive tobacco use presented with a dry cough and pleuritic chest pain. Further workup was significant for leukocytosis, macrocytic anemia, left lower lung mass, bilateral supraclavicular, hilar, and mediastinal lymphadenopathy. After a comprehensive rheumatologic workup was completed, the patient was found to have strongly positive antinuclear antibody (ANA) and negative scleroderma-specific antibodies with fluorescent ANA indicating a nucleolar pattern. We present a case of paraneoplastic scleroderma in the setting of lung adenocarcinoma which emphasizes the bidirectional relationship that exists between malignancy and rheumatic diseases.

## Introduction

Scleroderma is a well-documented autoimmune disease characterized by cutaneous fibrosis and multi-system involvement. The immunopathogenesis of this condition results from excessive activation of fibroblasts at the site of tissue injury and the resulting deposition of extracellular matrix proteins (ECM). Specifically, types I and III collagen are believed to orchestrate fibrosis, vascular damage, inflammation, and systemic autoimmunity in scleroderma [1].

Systemic sclerosis (SSc) can be classified into two main categories based on the extent of integumentary and visceral organ involvement. Limited scleroderma, or CREST syndrome (calcinosis, Raynaud phenomenon, esophageal dysmotility, sclerodactyly, and telangiectasia), is identified by sclerosis restricted to the patient’s hands, arms, and face. Diffuse scleroderma is a rapidly progressive form characterized by widespread skin involvement. Diffuse scleroderma can also be associated with dysfunction of the heart, lungs, kidneys, and gastrointestinal tract [2]. One of the most catastrophic results of SSc is associated malignancy. SSc resulting in malignancy is well documented and the largest meta-analysis conducted by Bonifazi et al. found that patients with scleroderma had a relative risk of 1.75 of developing malignancy [3].

In recent literature, there has been a significant emphasis on further understanding the relationship between autoimmunity and malignancy. It has been postulated that there is a complex bidirectional relationship that exists between scleroderma and cancer. The details of this proposed interdependence have not been fully elucidated and suggest a paraneoplastic mechanism of acquired autoimmunity. It has been suggested that the release of cytokines by tumor cells causes a cross-reactivity and deposition of immune complexes in neighboring and distant tissue. This results in a loss of immune tolerance via the expression of auto-antigens and the formation of auto-antibodies. These antibodies then precipitate the development of paraneoplastic rheumatic syndromes (PRS) [4]. Moreover, after a detailed review of PRS cases, it has been shown that the rheumatological expression of malignancy typically follows or predates the discovery of cancer. In patients with suspected PRS treatment of the underlying cancer is associated with the resolution of the autoimmune disease.

In the presented vignette we discuss the clinical course of a patient with a case of PRS. The patient carried a diagnosis of SSc with negative specific antibodies and was later discovered to have lung adenocarcinoma.

## Case presentation

The patient is a 42-year-old female with a past medical history of scleroderma (diagnosed at age 31), hyperhomocysteinemia, bilateral posterior cerebral artery stroke, and a 20-pack-year history of tobacco use who presented to the emergency department complaining of a dry cough and pleuritic chest pain for one week. On a physical exam, she had facial and extremity telangiectasias, sclerodactyly, and prominent P2 heart sound with diminished breath sounds in the bases of her lungs bilaterally.

**Figure 1:**
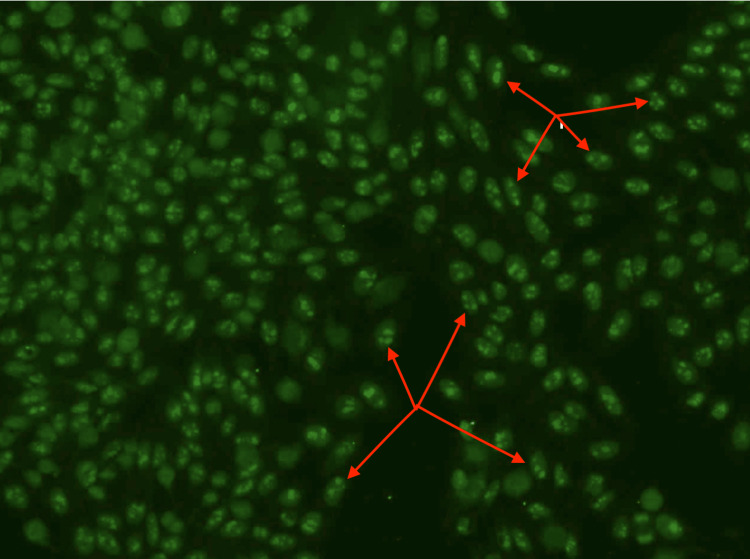
Immunofluorescent Staining of ANA Figure 1 demonstrates an image of the immunofluorescent staining of the pt's ANA indicating a nucleolar pattern. This can be best appreciated by the red arrows drawing attention to the circular fluorescence of the cells' nuclei. ANA: antinuclear antibody

The initial workup was significant for mild leukocytosis and macrocytic anemia with an unremarkable chemistry panel. Comprehensive autoimmune testing resulted and was negative, with the exception of a strongly positive antinuclear antibody (ANA) at a titer of 1:640. Reflex immunofluorescence staining demonstrated a nucleolar pattern (Figure 1).

**Figure 2:**
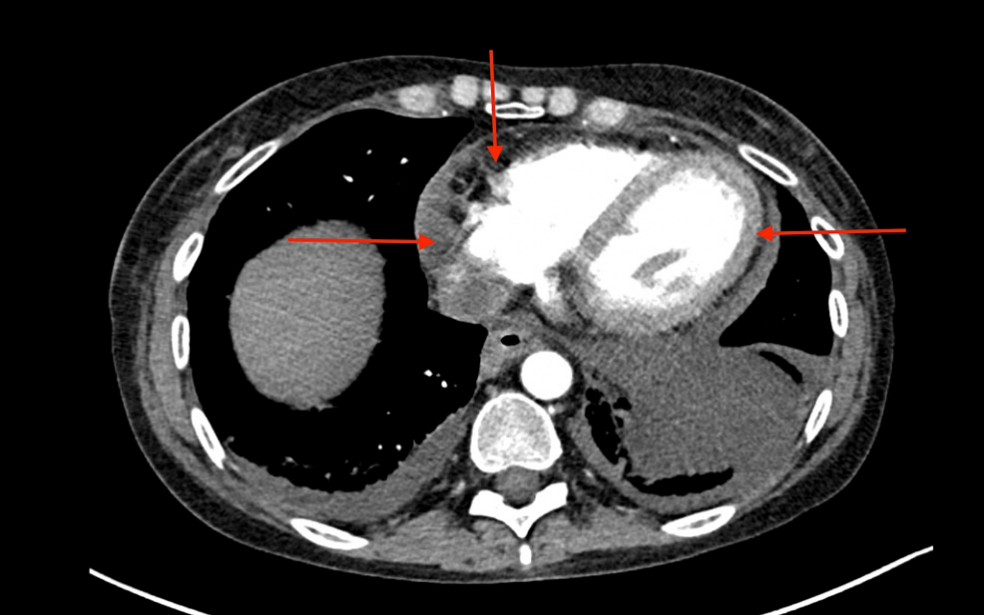
Computerized Tomography of Chest with Contrast Computerized tomography of the chest obtained during the patient's admission demonstrating a moderate-sized pericardial effusion.

Computed tomography (CT) chest demonstrated a left lower lung mass, bilateral supraclavicular, hilar, and mediastinal lymphadenopathy with diffuse honeycombing and paraseptal emphysema. Additionally, there were two hepatic lesions and a mild to moderate-sized pericardial effusion that demonstrated an interval increase in size when compared to previous imaging studies (Figure 2). Subsequent endobronchial ultrasound (EBUS) guided biopsy of the lesion was performed and pathology indicated stage 2b adenocarcinoma of the left lung.

## Discussion

Scleroderma is a clinical diagnosis that can be confirmed based on the European League Against Rheumatism (EULAR) and the American College of Rheumatology (ACR) criteria. Using this scoring system, a patient is classified as having SSc with a total number of points of nine or greater. In the presented case, the patient had clinical findings of Raynaud's, facial and extremity telangiectasias, sclerodactyly, calcinosis cutis, pulmonary fibrosis, interstitial lung disease, and a large symptomatic pericardial effusion. Based on the EULAR/ACR classification criteria the presented patient had a total score of 13 and thus the diagnosis of SSc was confirmed.

Prior to this hospitalization, the patient endorsed being previously diagnosed with scleroderma at an outside hospital. Despite being diagnosed with scleroderma based on clinical findings, the comprehensive rheumatologic workup including SSc-specific antibodies was negative except for ANA (Table 1). Positive scleroderma-related antibodies are neither sensitive nor specific for SSc, but in certain cases, they can assist in diagnosis when clinical findings are insufficient. In this case, the patient had significant manifestations of the syndrome, and the serological workup raised questions as to the inciting cause of the patient's autoimmune condition.

**Table 1:** Comprehensive Rheumatologic Panel Comprehensive rheumatologic panel indicating positive antinuclear antibody with negative systemic sclerosis-specific antibodies.

Component	Value	Ref Range and Units	Status
Anti-Nuclear Ab by IFA (RDL)	Positive!	Negative	Final
Anti-Centromere Ab (RDL)	<1:40	<1:40	Final
Anti-RNA Polymerase III (RDL)	<20	<20 Units	Final
Anti-SCL-70 Ab (RDL)	<20	<20 Units	Final
Anti-U1 RNP Ab (RDL)	<20	<20 Units	Final
Anti-U3 RNP Ab (Fibrillarin) (RDL)	Negative	Negative	Final
Anti-Th/To Ab (RDL)	Negative	Negative	Final
Anti-PM/SCL-100 AB (RDL)	<20	<20 Units	Final
Anti-PM/SCL-75 Ab (RDL)	<20	<20 Units	Final

In 2010 the ACR published that indirect immunofluorescence is the gold standard for ANA screening. In the presented case, the patient had an ANA analysis performed by indirect immunofluorescence which resulted in the staining of ANA in a nucleolar pattern. This staining pattern has consistently shown an association with the presence of malignancy and a poor prognosis if malignancy is identified. Moreover, in a recent study published by the University Hospital of Geneva, it was reported that the relative risk of associated malignancy in the context of ANA stained in a nucleolar pattern was 1.5 [5,6].

When considering the patient’s clinical presentation, laboratory results, and imaging findings it is postulated that the positive ANA titers were a result of a vigorous response by the patient’s immune system to her underlying lung adenocarcinoma. Due to the lack of previous hospital records, it is believed that this association was never investigated due to an incomplete diagnostic workup. For over a decade, our patient had been diagnosed with SSc which acutely resulted in severe clinical manifestations. Upon further investigation including immunofluorescence and imaging studies, it was concluded that the patient's untreated malignancy had resulted in a PRS.

A pressing question that arises from this case vignette is how clinicians will be able to differentiate PRS versus primary malignancy with concomitant rheumatologic disease. We suggest that in cases of rheumatological conditions that do not respond to the standard of care, despite aggressive management, there should be a comprehensive assessment for underlying malignancy. For health systems that have access to advanced screening methods, such as reflex immunofluorescence, this may be a key initial step in drawing this distinction. Moreover, if the fluorescent staining results in a nucleolar pattern, as in this case, we suggest that the given refractory rheumatological condition is likely the result of malignancy. If a clinician at this time has high enough suspicion for this complex process at play they should not hesitate to further investigate underlying malignancies associated with their patient's risk factors. In cases where malignancy is observed, our team recommends a multidisciplinary approach and treatment of the neoplastic process. We suspect that in patients who are treated, there will be a concomitant resolution of their rheumatologic condition. Unfortunately, due to several complications in our presented patient's clinical course she was unable to receive appropriate therapy for her malignancy and as a result, the resolution of her underlying rheumatological condition was not appreciated.

## Conclusions

This unique case sheds light on the relationship between malignancy and scleroderma and suggests the influence that cancer may play in developing autoimmune diseases. Although there has been convincing evidence of autoimmune disease resulting in various malignancies, cases such as the one presented represent the importance of further investigating a bidirectional relationship. Moreover, the distinct ANA fluorescent staining, in this case, signifies that a comprehensive workup for malignancy may be indicated in cases of PRS that are refractory to standard treatment. PRS, although uncommon, represent significant diagnostic and clinical challenges for providers across multiple specialties. As a result, we urge further investigation into this topic in the hopes of expanding medicine’s understanding of the complex bidirectional relationship that exists between malignancy and rheumatic disease.
